# Complementary structure of statistical significance and predictive relevance in explainable machine learning–based transcriptomic tissue classification of Hanwoo cattle

**DOI:** 10.3389/fgene.2026.1818099

**Published:** 2026-07-06

**Authors:** Dogyeong Lee, Junyoung Lee, Inchul Choi, Dajeong Lim

**Affiliations:** 1 Department of Bio-Big Data, Chungnam National University, Daejeon, Republic of Korea; 2 Department of Bio-AI Convergence, Chungnam National University, Daejeon, Republic of Korea; 3 Division of Animal and Dairy Sciences, College of Agriculture and Life Sciences, Chungnam National University, Daejeon, Republic of Korea

**Keywords:** differential gene expression, feature attribution, Hanwoo cattle, machine learning, RNA sequencing, shapley additive explanations, tissue classification

## Abstract

Understanding tissue-specific transcriptomic structures in livestock is essential for elucidating the molecular basis of economically important traits. Conventional differential gene expression analysis efficiently identifies genes with large average expression differences but does not fully capture multivariate expression structures and gene-gene interaction patterns that define tissue identity. In this study, we developed an explainable machine learning framework to classify seven Hanwoo cattle tissues using RNA sequencing data and to systematically compare the relative contributions of statistical and model-derived signals. A Random Forest–based one-versus-rest classification model was trained on 130 Hanwoo transcriptomes and externally validated using 231 independent *Bos taurus* samples derived from heterogeneous public datasets following reference-based batch correction. Repeated balanced validation demonstrated stable generalization performance, achieving a mean accuracy of 0.907 and a macro-average area under the receiver operating characteristic curve of 0.963. A comparative analysis of gene sets selected by differential expression analysis, genes prioritized by model-based feature attribution, their union, and randomly selected genes within the same classification framework revealed that strongly differentially expressed genes form the primary discriminatory structure for tissue classification. In contrast, integration of model-prioritized genes enhanced classification performance, particularly for biologically related tissues, whereas randomly selected genes produced reduced and unstable predictive performance. Model interpretation further revealed that highly contributory genes were consistent with known tissue-specific biological functions and exhibited non-linear, expression-dependent contribution patterns shaped by coordinated multigene contexts. These findings indicate that tissue identity is supported by a hierarchical transcriptional structure in which dominant differential signals establish primary class boundaries and multivariate interaction patterns refine decision surfaces. The proposed framework provides an interpretable strategy for distinguishing statistical significance from predictive relevance in high-dimensional transcriptomic data and offers practical implications for molecular marker development in livestock genomics and breeding programs.

## 1 Introduction

Hanwoo (*Bos taurus coreanae*) is an indigenous cattle breed of the Korean Peninsula and occupies a central position in the domestic livestock industry due to its superior meat quality and high economic value (Chung et al., 2018). Growth performance and carcass-related traits in Hanwoo are regulated by interactions among multiple genes and biological pathways related to growth and lipid metabolism (Strucken et al., 2017), and these traits have been reported to be closely associated with molecular networks involved in growth and lipid metabolism (Naserkheil et al., 2020). Accordingly, elucidating tissue-level functional characteristics and identifying tissue-specific gene expression patterns have been recognized as critical research objectives for establishing effective Hanwoo breeding strategies and improving livestock productivity (Naserkheil et al., 2020). In particular, advances in RNA-seq have improved quantification of gene expression across tissues and enabled tissue-specific transcriptome analysis, enhancing the resolution of livestock genomics research (Wang et al., 2009; Finotello and Di Camillo, 2015).

Conventionally, statistical differential gene expression (DEG) analysis has been a core approach for identifying tissue-specific genes, and it has been widely used to select genes that are preferentially expressed in specific tissues through comparative analyses of expression levels across multiple tissue types (Liang et al., 2006). However, DEG-based approaches rely on univariate statistical tests that focus on differences in average expression levels, which limits their ability to adequately capture complex transcriptomic characteristics such as gene–gene interaction structures, multimodal expression distributions, and non-linear expression patterns (Wang et al., 2019). To address these limitations, machine learning and deep learning models have recently emerged as powerful tools for improving tissue classification and prediction performance by learning non-linear expression patterns and latent representations from high-dimensional omics data.

In human transcriptomic research, tissue-specific gene expression patterns have been widely used to investigate disease mechanisms and identify potential biomarkers. Large-scale resources, such as the Genotype-Tissue Expression (GTEx) project, have enabled systematic profiling of gene expression across a wide range of human tissues. Subsequent studies have demonstrated that genetic risk for many complex diseases is enriched in genes with tissue-specific expression, highlighting disease-relevant tissues and cell types (Lonsdale et al., 2013; Finucane et al., 2018). Based on these findings, machine learning–based approaches have been increasingly applied to high-dimensional transcriptomic data to capture complex, multivariate expression patterns. These methods have facilitated the identification of predictive gene signatures associated with complex diseases, including cancer, metabolic disorders, and neurological conditions, thereby improving diagnostic and prognostic performance (Kourou et al., 2015; Libbrecht and Noble, 2015).

Machine learning (ML) frameworks are increasingly used in livestock transcriptomics to extract minimal gene subsets without sacrificing classification performance. The recent study compared traditional DEG methods with tree-based ML models (RF, XGBoost) and their hybrid (RX) using bovine multi-tissue data. The results demonstrated that the hybrid ML approach achieved the highest average accuracy while utilizing a more parsimonious gene set compared to conventional methods (Chen et al., 2021).

Tissue-specific gene expression analyses in livestock have mainly served as an interpretative framework for investigating the molecular basis of economically important traits. Comparative transcriptomic analyses between muscle and adipose tissues in cattle have identified tissue-enriched regulatory programs associated with muscle development and fat deposition (Choi et al., 2019). In addition, comprehensive multi-tissue expression atlases have been established to systematically characterize tissue- and cell-type–specific transcriptional landscapes, providing reference resources for linking complex traits to biologically relevant tissues (Han et al., 2025).

Furthermore, genomic prediction studies in beef cattle have shown that ML and deep learning models, including Random Forest, multilayer perceptrons, and convolutional neural networks, can effectively capture non-linear genetic architectures underlying growth-related traits, highlighting their suitability for modeling complex biological relationships in high-dimensional datasets (Hay, 2024). In addition, recent reviews in animal breeding have emphasized the increasing adoption of ML-based genomic prediction frameworks as complementary tools to conventional linear models for handling epistatic effects and non-additive genetic variation (Chafai et al., 2023). Nevertheless, most machine learning and deep learning models exhibit “black-box” characteristics, in which the internal decision-making processes are not intuitively interpretable by humans. This lack of transparency has been identified as a major limitation for establishing trust in model predictions, enabling biological interpretation, and facilitating practical applications in the life sciences (Guidotti et al., 2018).

Recent studies have demonstrated the increasing application of machine learning approaches in livestock transcriptome analysis. For example, machine learning models combined with differential expression analysis and WGCNA have been applied to bovine muscle transcriptome datasets to identify candidate genes associated with muscle growth and lipid metabolism (Li et al., 2025). These approaches integrated multiple analytical methods and used SHAP-based interpretation to prioritize biologically meaningful genes from large-scale RNA-seq data. In addition, recent studies have highlighted the potential of deep learning and machine learning models for genomic prediction and functional interpretation in animal breeding, demonstrating their ability to capture complex nonlinear relationships and improve gene prioritization from high-dimensional transcriptomic datasets (Montesinos-Lopez et al., 2021). These findings support the applicability of machine learning-based frameworks for tissue-specific gene classification and functional interpretation in livestock transcriptomic studies.

**FIGURE 1:**
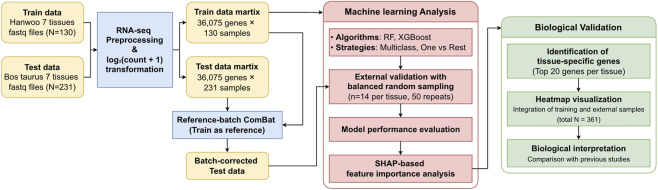
Overall workflow of the study.

In this study, we developed a tissue classification framework that, while demonstrated using Hanwoo incorporates Explainable Artificial Intelligence (XAI) as a versatile methodology applicable to various livestock species. XAI has been proposed as an approach that enhances both model transparency and practical applicability by providing human-interpretable explanations of the decision-making process (Gunning and Aha, 2019). To this end, we constructed a Random Forest (RF)-based One-vs-Rest (OvR) classification model, which is known to achieve high classification performance and stable generalization capability. RF integrates multiple decision trees using an ensemble strategy, thereby reducing overfitting while maintaining strong predictive performance and reliable feature importance estimation in high-dimensional feature spaces (Breiman, 2001). In addition, we applied SHapley Additive exPlanations (SHAP) to quantitatively evaluate the contribution of individual genes to tissue classification predictions. SHAP is a feature attribution method based on Shapley values from cooperative game theory and has been proposed as a unified interpretation framework that simultaneously satisfies key properties, including local accuracy, consistency, and missingness (Lundberg and Lee, 2017b). Through this integrated approach, the present study aims to preserve model predictive performance while systematically identifying tissue-specific genes that play critical roles in tissue classification and strengthening the biological interpretability of these features, thereby improving both the reliability and explainability of the proposed framework. The overall workflow of this study is presented in Figure 1.

## 2 Materials and methods

### 2.1 Data preparation

#### 2.1.1 Train dataset

Tissue samples were collected *postmortem* from 22 Hanwoo males and steers raised at the Hanwoo Cattle Research Institute, National Institute of Animal Science (NIAS), Republic of Korea, under standardized feeding and management conditions. To minimize physiological variation among individuals, all animals were fasted for 24 h prior to slaughter under identical resting conditions. Sample collection was conducted between August 2020 and February 2024. The age of the animals at slaughter was recorded (mean ± SD, 19.2 ± 9.1 months). The animals covered a broad age range (approximately 6–30 months), including younger intact males and older steers representing typical production stages in Hanwoo cattle. This reflects common management practices in Hanwoo production systems, where animals are raised and finished across different physiological stages. All experimental procedures were reviewed and approved by the NIAS Animal Ethics Committee (approval number: NIAS20201979).

A total of 130 RNA-seq transcriptome samples were generated from seven tissues, including Abdominal Fat, Back Fat, Blood, Liver, Longissimus, Rumen, and Tenderloin. Multiple tissues were collected from each animal, resulting in a multi-tissue transcriptome dataset representing individual-level variation. To account for potential genetic bias, the animals were derived from a heterogeneous paternal background. According to pedigree records, the 22 animals represented 13 different sire IDs, with most sires shared by only two to three individuals. Because the training dataset consisted exclusively of purebred Hanwoo animals and all recorded sires were also Hanwoo, breed-level paternal effects could not be evaluated in the present study. Accordingly, the paternal factor considered in the current analysis was sire ID rather than sire breed. This multi-sire composition ensures that the identified transcriptomic signatures reflect tissue-specific patterns rather than lineage-specific variations. This dataset was used as the training dataset for model construction.

The raw FASTQ files have been deposited in the NCBI Sequence Read Archive (SRA) under BioProject accession number PRJNA1426915. Detailed sample information, including SRA run ID, BioProject, BioSample accession, tissue type, sequencing platform, and library layout, is provided in Supplementary Table S1.

#### 2.1.2 Test dataset for generalization performance validation

To rigorously assess the generalization capability of the developed model, publicly available *B. taurus* RNA-seq data, deposited in the NCBI Sequence Read Archive (SRA), were utilized. Specifically, the FASTQ files employed in this analysis originated from the following BioProject accessions: PRJNA171257, PRJNA393239, PRJNA419520, PRJEB94322, PRJNA824614, PRJNA1002884, and PRJNA1112730. This external dataset consisted of 231 samples from abdominal adipose tissue, omental fat tissue, subcutaneous backfat tissue, whole blood, liver, Longissimus dorsi muscle, rumen, and psoas major (PM). These external samples were classified into the same seven tissue categories as the training dataset for final performance evaluation, with detailed sample information provided in Supplementary Table S2. The number of samples included in the train and test datasets is summarized in Table 1.

**TABLE 1:** Number of samples in train and test datasets.

Tissue	Train dataset (N)	Test dataset (N)
Abdominal fat	18	14
Back fat	20	22
Blood	20	39
Liver	18	36
Longissimus	18	45
Rumen	18	61
Tenderloin	18	14

### 2.2 RNA-seq preprocessing pipeline

#### 2.2.1 Quality control and filtering

Quality control of raw RNA-seq data was performed using FastQC (v.0.12.1; Babraham Bioinformatics, Cambridge, UK) (Andrews et al., 2010), and summary reports were generated using MultiQC (v.1.18; Babraham Bioinformatics, Cambridge, UK). Adapter trimming and quality filtering of paired-end reads were conducted using Trimmomatic (v.0.39; Usadel Lab, Cambridge, UK) (Bolger et al., 2014) in paired-end (PE) mode. The following parameters were applied: adapter clipping (ILLUMINACLIP:TruSeq3-PE.fa:2:30:10), trimming of low-quality bases from read ends (LEADING:3, TRAILING:3), filtering using a sliding window approach with a 4-bp window and a minimum mean quality score of 15 (SLIDINGWINDOW:4:15), and retention of reads with a minimum length of 36 bp (MINLEN:36). This process ensured the removal of adapter sequences, low-quality bases due to sequencing errors, and short reads.

#### 2.2.2 Genome alignment and read counting

Filtered reads were aligned to the *B. taurus* reference genome ARS-UCD1.3 using HISAT2 (v2.2.1; Center for Computational Biology, Johns Hopkins University, Baltimore, MD, USA) (Kim et al., 2019) with default parameters optimized for spliced alignment. The resulting alignment files were generated in SAM format and subsequently converted to sorted BAM files using SAMtools (v1.22; Wellcome Sanger Institute, Cambridge, UK) for downstream analyses.

Gene-level read quantification was performed using featureCounts (v2.1.1; Subread package) (Liao et al., 2014) based on the Ensembl Bos_taurus.ARS-UCD1.3.112. gtf annotation file (Ensembl release 112). Only uniquely mapped reads assigned to annotated exonic regions were counted. This workflow generated a raw count matrix comprising 36,075 annotated genes, which was subsequently used for differential expression analysis and machine learning–based tissue classification.

### 2.3 Machine learning data transformation and preprocessing

#### 2.3.1 Data normalization and transformation and batch effect correction

Gene expression counts were log_2_ (count +1) transformed to adjust for library size differences and stabilize variance, following standard RNA-seq preprocessing practices (Anders and Huber, 2010; Love et al., 2014). This ensured numerical stability and reduced data skewness, improving suitability for classification model.

Batch effects, which arise from technical variations such as differences in sequencing platforms, laboratory protocols, reagent lots, and processing conditions, can introduce substantial non-biological variability into transcriptome data and significantly impair the generalization performance of predictive models (Leek et al., 2010). The standard ComBat algorithm corrects batch effects by estimating batch-specific location and scale parameters using an empirical Bayes framework, which involves jointly adjusting all batches and may consequently alter the marginal distribution of the training data, particularly when biological factors are partially confounded with batch structure (Johnson et al., 2007). To mitigate this issue, we adopted a reference-batch ComBat strategy, which preserves the statistical properties of the training dataset by fixing the reference batch and aligning only the non-reference batches to the reference distribution, thereby preventing distortion of the training data and reducing information loss (Van et al., 2024). This approach has been shown to improve cross-study prediction performance by maintaining distributional consistency between training and external test datasets (Van et al., 2024). In the present study, the log-transformed Hanwoo transcriptome dataset was designated as the reference batch, and batch effect correction was applied exclusively to the log-transformed external bovine transcriptome dataset. All batch effect adjustments were performed using the ComBat function implemented in the sva R package (v3.54.0), which provides a standardized framework for empirical Bayes-based batch correction and removal of unwanted technical variation in high-throughput genomic data (Johnson et al., 2007; Leek et al., 2012).

### 2.4 Classification model construction and evaluation

#### 2.4.1 Machine learning model construction

Tissue classification models were developed using transcriptome datasets that had undergone comprehensive batch effect correction. Random Forest (RF) and Extreme Gradient Boosting (XGBoost) algorithms were employed as tree-based ensemble classifiers due to their strong performance in high-dimensional biological data and their ability to capture complex non-linear feature interactions (Breiman, 2001; Chen, 2016). RF constructs multiple decision trees using bootstrap aggregation and random feature selection, which improves prediction stability and reduces overfitting while providing intrinsic feature importance measures for model interpretation (Breiman, 2001). Although conventional RF importance metrics may be biased toward variables with specific data characteristics, permutation-based importance approaches have been shown to provide more reliable and statistically robust estimates of feature contributions (Strobl et al., 2007; Altmann et al., 2010).

XGBoost implements gradient boosting with regularization and optimized tree construction strategies, enabling scalable learning and state-of-the-art predictive performance across complex classification tasks (Chen, 2016). To ensure consistent and theoretically grounded feature attribution for both RF and XGBoost models, tree-based SHAP (TreeSHAP) was used to quantify feature contributions, providing exact Shapley value explanations with guarantees of local accuracy and consistency (Lundberg et al., 2020). Furthermore, permutation-based importance measures have been widely adopted as a general framework for assessing variable reliance across different model classes, improving the robustness and interpretability of predictive models (Fisher et al., 2019).

All computational analyses were conducted in a Python (v.3.13.5) environment, with RF models implemented using the scikit-learn package (v1.7.2) and XGBoost models constructed using the xgboost library (v3.1.2).

To address the multi-tissue classification problem, a multiclass classification approach was combined with a One-vs-Rest (OvR) strategy. Multiclass classification models were trained as a single integrated model for each algorithm. For the OvR strategy, individual binary classifiers were constructed for each tissue by setting it as the positive class, resulting in a total of seven binary classifiers. Upon completion of training, all models were individually saved in joblib (v1.5.2) format for subsequent reuse in the external validation phase.

#### 2.4.2 External validation and iterative sampling-based prediction

An independent *B. taurus* transcriptome dataset served as the external test set for evaluating model generalization. Gene features were first matched between training and external datasets to ensure consistent input dimensions. Expression values were then transformed using log_2_ (count +1) scaling and adjusted through reference-batch ComBat normalization. Because tissue sample sizes differed across external datasets, a balanced evaluation strategy was adopted. For each validation run, the number of samples per tissue was restricted to the smallest available class (n = 14). Equal-sized subsets were randomly drawn from each tissue category. This resampling procedure was performed 50 times to obtain stable performance estimates. Classification outcomes were summarized at both tissue-specific and overall levels. Macro-averaged metrics across the seven tissue classes were used to reduce bias arising from class imbalance and variable classification difficulty. This metric was calculated by taking the arithmetic mean of the individual accuracies obtained from each of the seven tissue-specific binary classifiers, ensuring that each tissue type contributed equally to the final performance score (Alanazi and Alanazi, 2025).

$${Accuracy}_{Multiclass\;Classification}=\frac{1}{N}\sum_{i=1}^{N}1\left({\hat{y}}_{i}=y_{i}\right)$$

where *N* is the number of samples in the test dataset, ${\hat{y}}_{i}$ and ${\;y}_{i}$ respectively represent the true and predicted class labels of sample i and $1\left(\cdot \right)$ is the indicator function.

$${Accuracy}_{Binary\;Classification}=\frac{TP+TN}{TP+TN+FP+FN}$$

where TP, TN, FP, and FN are respectively the numbers of true positives, true negatives, false positives, and false negatives (Bishop and Nasrabadi, 2006).

Model performance was quantified using classification accuracy and the area under the receiver operating characteristic curve (AUC-ROC). Accuracy indicates the proportion of correctly assigned tissue labels, whereas AUC reflects the model’s ability to separate tissue classes across different decision thresholds. Performance values obtained from repeated resampling were combined to generate a macro-averaged ROC curve, which provides an integrated summary of multi-tissue classification performance.

#### 2.4.3 Comparative evaluation of feature selection strategies

To assess whether the predictive performance of the machine learning was primarily driven by large univariate expression differences between tissues, we conducted feature-set specific performance comparison using four distinct gene selection strategies. The four models were constructed as follow under a consistent RF-OvR classification framework.

- Model 1 (DEG-only): A DEG-only feature set was constructed by selecting the top 20 DEGs from each One-vs-Rest tissue comparison, based on criteria of |log_2_ fold change| > 1 and a false discovery rate (FDR) < 0.05. For each tissue, DEGs were ranked by ascending FDR, and the top 20 genes were selected. DEG lists from all seven tissues were subsequently combined. Because overlapping genes across tissues were retained, the total number of features was 133.
- Model 2 (SHAP-only): A SHAP-only feature set was constructed by selecting the top 20 genes per tissue based on mean absolute SHAP values derived from the RF-OvR models. To exclude genes identified primarily by univariate differential expression, only SHAP-prioritized genes that did not satisfy the DEG significance criteria were retained. SHAP-selected gene lists from all seven tissues were then combined, allowing overlap across tissues, resulting in 79 features.
- Model 3 (Union): A combined feature set was constructed by taking the union of the DEG-only and SHAP-only gene sets, thereby integrating 212 genes identified by both statistical differential expression and model-derived feature attribution.
- Model 4 (Random): A random feature set was used as a control to estimate baseline classification performance. For each iteration, 140 genes were randomly sampled from the full expression matrix, matching the feature set size of the DEG- and SHAP-based models. This random sampling procedure was repeated 50 times to obtain stable estimates of performance variability.

For all feature sets classification models were trained using the same framework and hyperparameters to ensure comparability. Model performance was evaluated on the same independent external test dataset used in the primary model evaluation, using repeated balanced sampling (14 samples per tissue, 50 iterations). Although the models were trained in a One-vs-Rest binary framework, final tissue labels were determined using an argmax decision rule across the seven classifiers, assigning each sample to the tissue with the highest predicted probability. Performance metrics included the macro-averaged F1 score and confusion matrices aggregated across iterations. Macro-F1 was selected as the primary evaluation metric for feature-set comparison due to its robustness under balanced multiclass resampling.

### 2.5 Model interpretation and biological validation

#### 2.5.1 SHAP-based feature importance analysis

For model interpretation, the Random Forest-based One-vs-Rest (RF OvR) model, which demonstrated superior performance in external validation, was selected as the final interpretative model. Given that RF OvR consists of binary classifiers for each tissue, the seven corresponding joblib binary models were individually loaded to perform SHAP (SHapley Additive Explanations) analysis. A SHAP-based TreeExplainer was applied to quantify each gene’s contribution to the prediction outcome of the trained RF model for a specific tissue type. The SHAP analysis was conducted in a Python environment using the SHAP library (v0.49.1), loading the joblib files of the RF classifiers generated during training. After calculating SHAP values, which represent the gene (feature) contribution to a sample’s prediction in each tissue-specific model, these values were converted to absolute magnitudes. The Mean Absolute SHAP Value was then computed by averaging these absolute values across both the class and sample axes. Based on these metrics, genes were ranked by importance for each tissue, and the top 20 genes were defined as tissue-specific key predictive genes, subsequently used for expression pattern visualization and literature-based biological validation.

#### 2.5.2 Comparative analysis with DEGs

To compare the results of SHAP-based gene importance with traditional statistical differential expression analysis, DEG analysis was performed exclusively on the Hanwoo transcriptome dataset (training data). Differential expression analysis was conducted using DESeq2 (v1.46.0) in a one *versus* rest comparison manner for each tissue against all other tissues. Genes were selected as differentially expressed based on a false discovery rate (FDR) < 0.05 and |log_2_ fold change| > 1. This process aimed to identify sets of genes with statistically significant expression changes specific to each tissue and to compare their concordance and distinctiveness with the SHAP-based important genes.

#### 2.5.3 Integrated expression pattern visualization

To visualize tissue-specific expression patterns of SHAP-prioritized genes, a combined expression matrix was constructed using log-transformed values from both training and external datasets. The final matrix contained expression profiles from 361 samples aligned to a unified gene feature set. Gene-wise Z-score normalization was applied to standardize expression levels and enable relative comparisons across tissues. Heatmaps were generated using the pheatmap R package (v1.0.13). Hierarchical clustering based on Euclidean distance was performed for both genes and samples to examine global expression structure and tissue-level grouping patterns.

#### 2.5.4 SHAP dependence plot analysis

For each tissue classifier, SHAP dependence plots were generated based on the top 20 genes with the highest SHAP importance values. The highest-ranked gene was selected as the primary feature and paired individually with the other SHAP-prioritized genes. This strategy was applied to examine variation in the contribution pattern of the primary gene under different expression conditions of the associated features. In addition, we compared these SHAP-based patterns with the upregulated and downregulated gene sets identified from DEG analysis to analyze trends between model-derived feature contributions and differential expression.

## 3 Results

### 3.1 RNA-seq data quality control and preprocessing

Quality control and preprocessing were conducted for the Hanwoo training dataset (n = 130) and the external cattle validation dataset (n = 231). Following adapter trimming and removal of low-quality bases, the majority of sequencing reads were retained for downstream analyses. The average clean read retention rate was 95.60% ± 4.05% for the Hanwoo dataset and 90.14% ± 11.53% for the external dataset. Alignment to the *B. taurus* reference genome (ARS-UCD1.3) using HISAT2 resulted in consistently high mapping rates across both datasets, with average values of 96.90% ± 3.38% for Hanwoo samples and 95.67% ± 4.44% for external *B. taurus* samples. After read counting and filtering, a unified gene expression matrix consisting of 36,075 genes across 361 samples was generated. The expression values were subsequently transformed using log_2_ (count +1) to reduce the influence of extreme values and improve numerical stability for machine learning analyses. Reference-batch ComBat normalization was then applied to adjust the external dataset toward the distribution of the Hanwoo training data, thereby reducing technical discrepancies between datasets while maintaining tissue-specific expression patterns.

### 3.2 Assessment of confounding factors on tissue-specific expression

**FIGURE 2:**
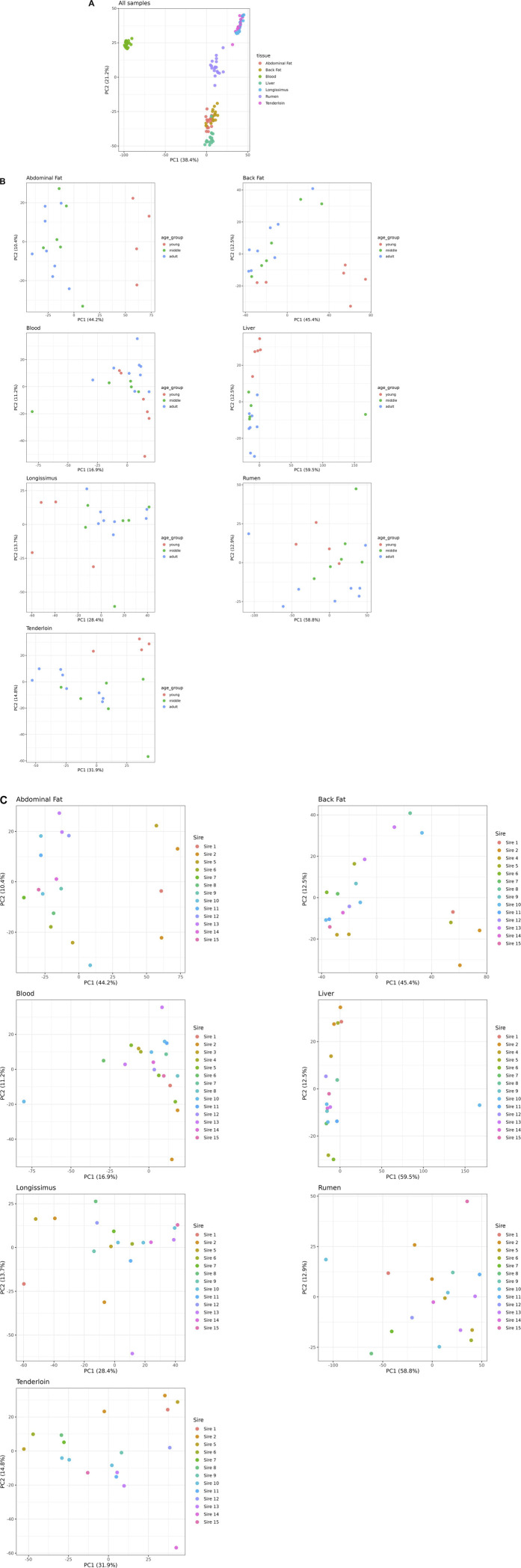
Principal component analysis (PCA) assessing potential confounding effects of age and sire background on tissue-specific transcriptomic patterns in the Hanwoo training dataset. **(A)** PCA of all samples based on the full expression matrix, colored by tissue type. **(B)** Tissue-wise PCA plots colored by age group, where samples were categorized as young (6–12 months), middle (18–22 months), and adult (26–30 months). **(C)** Tissue-wise PCA plots colored by sire background, with samples lacking sire information labeled as NA.

To evaluate whether the broad age range and paternal heterogeneity could confound tissue-specific transcriptomic patterns in the Hanwoo training dataset, we performed additional exploratory and statistical analyses, including principal component analysis (PCA), tissue-wise PERMANOVA, and dispersion testing. PCA based on the full expression matrix showed that samples were primarily separated according to tissue identity, indicating that tissue type was the major source of global transcriptomic variation (Figure 2A). Within individual tissues, PCA colored by age group did not show strong or consistent clustering according to age within most tissues (Figure 2B). Likewise, tissue-wise PCA colored by sire background, including samples with unavailable sire information labeled as NA, did not reveal clear clustering patterns attributable to sire effects (Figure 2C). These exploratory analyses suggest that neither age nor sire background was a dominant source of within-tissue transcriptomic variation.

Tissue-wise PERMANOVA further showed that age was not a significant driver of transcriptomic variation in most tissues, with the exception of back fat, where a significant but modest effect was detected (*R* ^2^ = 0.089, p-value = 0.040; Table 2). In contrast, sire background, evaluated at the level of sire ID within the purebred Hanwoo training dataset, was not significant in any tissue, suggesting that paternal heterogeneity did not act as a major global confounding factor in this dataset. Although age showed a significant association in back fat, the proportion of variance explained was limited, indicating that the effect of age was limited and that tissue-specific expression structure remained the predominant source of variation.

**TABLE 2:** Tissue-wise PERMANOVA summary of age and sire effects on transcriptomic variation in the Hanwoo training dataset.

Tissue	Age *R* ^2^	Age p-value	Sire *R* ^2^	Sire p-value
Abdominal fat	0.083	0.103	0.407	0.660
Back fat	0.089	0.040	0.546	0.194
Blood	0.042	0.811	0.576	0.873
Liver	0.070	0.577	0.420	0.909
Longissimus	0.072	0.151	0.579	0.386
Rumen	0.022	0.845	0.602	0.675
Tenderloin	0.092	0.079	0.539	0.620

*R* ^2^ values indicate the proportion of transcriptomic variance explained by each factor within each tissue. P-values were obtained using permutation-based testing.

To further assess whether the PERMANOVA results were influenced by heterogeneity of within-group dispersion, dispersion tests were additionally performed. Significant age-group dispersion was observed only in back fat, whereas the other six tissues did not show significant age-related dispersion heterogeneity (Supplementary Table S3). This pattern suggests that the back fat signal likely reflects biologically meaningful developmental variability in adipose tissue across age groups, rather than a pervasive confounding effect across the entire dataset. Taken together, these results indicate that tissue identity remained the dominant source of transcriptomic variation in the training dataset, while age-related effects were limited and tissue-specific, particularly in back fat. However, supplementary dispersion testing indicated significant sire-related heterogeneity in the two muscle tissues, and these tissues also showed less distinct classification in subsequent analyses, suggesting greater transcriptomic overlap within the present analytical framework.

### 3.3 Model performance and generalization on independent external dataset

Comparative analysis of classification performance across different model configurations demonstrated that both the learning algorithm and classification strategy had a substantial impact on predictive accuracy and AUC. As summarized in Table 3, evaluations using the external validation dataset showed that Random Forest (RF)-based models consistently achieved higher mean accuracy and mean AUC values than Extreme Gradient Boosting (XGBoost)-based models.

**TABLE 3:** Comparison of mean performance metrics for tissue classification.

Model configuration	Mean accuracy	Mean AUC
RF - multiclass	0.790 ± 0.019	0.971 ± 0.001
RF - OvR	0.907 ± 0.004	0.963 ± 0.004
XGB - multiclass	0.728 ± 0.021	0.934 ± 0.006
XGB - OvR	0.796 ± 0.015	0.922 ± 0.007

Moreover, the application of the One-vs-Rest (OvR) strategy led to improved classification performance across both algorithms compared to the multiclass approach. Across the four evaluated model configurations, RF-OvR achieved the highest classification accuracy (mean = 0.907 ± 0.004) across 50 repeated evaluations.

While the RF-multiclass model achieved the highest AUC (0.971 ± 0.001), it showed lower classification accuracy (0.790 ± 0.019) compared to RF-OvR, which balanced both metrics. In contrast, RF-OvR increased classification accuracy by approximately 11.7 percentage points while maintaining a high discriminative performance (AUC = 0.963 ± 0.006).

**FIGURE 3:**
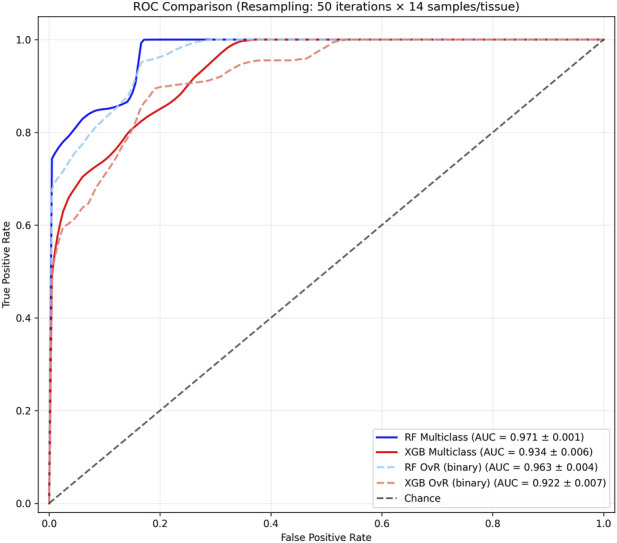
AUC-ROC analysis of tissue classification models on the external dataset.

AUC-ROC curve analysis using an independent external dataset further confirmed the generalization capability of the RF-OvR model (Figure 3). When 14 samples per tissue were randomly selected and evaluated across 50 repeated iterations, the macro-average AUC remained stable at 0.963 ± 0.006. Based on these results, RF-OvR was selected as the final model for subsequent gene prioritization analyses.

### 3.4 Comparison of classification performance across different feature sets

To assess the impact of different gene selection strategies on tissue classification performance, we compared four models: Model 1 (DEG-only), Model 2 (SHAP-only), Model 3 (Union), and Model 4 (Random).

**FIGURE 4:**
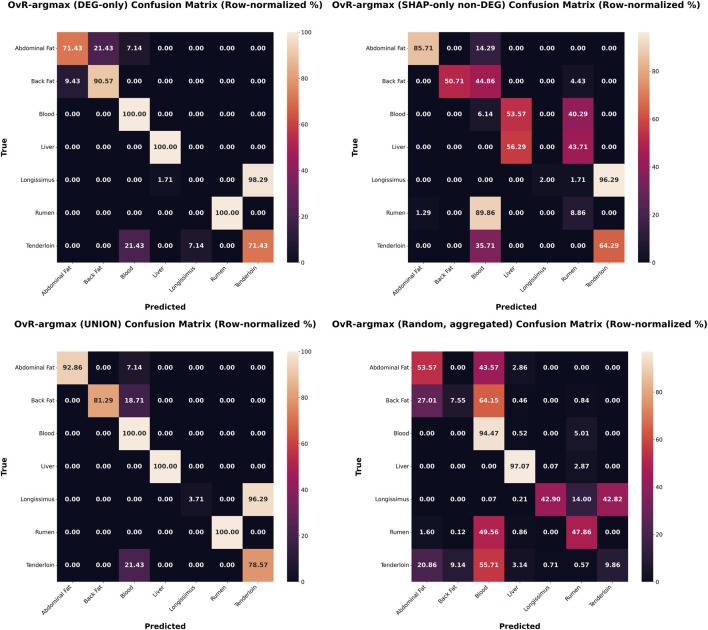
RF-OvR-Argmax Multiclass Confusion Matrices Across Different Feature Sets. Row-normalized confusion matrices (percentage per true class) are shown for the DEG-only, SHAP-only (non-DEG), Union, and size-matched random gene sets. Predictions were generated using Random Forest one-vs-rest classifiers, and final class labels were assigned based on the highest predicted probability (argmax). Confusion matrices were aggregated across 50 balanced sampling iterations.

The Model 1 achieved an accuracy of 0.762 ± 0.007 and a macro-F1 score of 0.720 ± 0.007 (Table 3). The confusion matrix indicated strong classification performance for Blood, Liver, and Rumen tissues. Adipose tissues (Abdominal Fat and Back Fat) were largely distinguishable, although minor cross-classification was observed. In contrast, muscle tissues showed substantial misclassification (Figure 4). Longissimus was frequently predicted as Tenderloin, and 21.43% of Tenderloin samples was predicted as Blood. The Model 2 achieved an accuracy of 0.391 ± 0.020 and a macro-F1 score of 0.397 ± 0.019. The confusion matrix indicated markedly reduced classification performance across all tissue types. Most samples were predominantly predicted as Blood, resulting in poor discrimination among the seven tissues overall. The reduced performance of the SHAP-only (non-DEG) model reflects that SHAP-identified features operate primarily as interaction-refining signals rather than primary discriminative drivers. Removal of strong univariate DEGs reduces the dominant class-separating structure, leading to diminished standalone predictive power. The Model 3 achieved the highest performance among the four feature selection strategies, with an accuracy of 0.795 ± 0.012 and a macro-F1 score of 0.758 ± 0.016. The confusion matrix indicated improved separation between Abdominal Fat and Back Fat compared with the DEG-only model. However, discrimination between muscle tissues remained limited, with persistent confusion involving Longissimus and Tenderloin. This pattern suggests that, although tissue-specific signals were present, the two muscle tissues remained less separable than other tissue categories within the present classification framework. The Model 4 achieved an accuracy of 0.505 ± 0.089 and a macro-F1 score of 0.461 ± 0.092. The confusion matrix indicated inconsistent and unstable classification across tissue types, with no clear tissue-specific discrimination pattern. These results suggest that classification performance was not driven by feature number alone. The classification performance of the DEG-only, SHAP-only, union, and random feature sets is summarized in Table 4.

**TABLE 4:** Argmax multiclass performance comparison across feature selection strategies**.**

Model	Feature strategy	Number of features	Accuracy (mean ± SD)	Macro-F1 (mean ± SD)
Model 1	DEG-only	133	0.762 ± 0.007	0.720 ± 0.007
Model 2	SHAP-only (non-DEG)	79	0.391 ± 0.020	0.397 ± 0.019
Model 3	DEG ∪ SHAP (union)	212	0.795 ± 0.012	0.758 ± 0.016
Model 4	Random	140	0.505 ± 0.089	0.461 ± 0.092

Values are presented as the mean ± standard deviation (SD) derived from 50 repeated balanced sampling iterations, with evaluations conducted on an independent external test dataset.

Classification accuracy remained high even when relatively compact gene subsets were used. The DEG-only model maintained strong performance with 133 genes, suggesting that tissue-discriminative transcriptional signals are concentrated within a limited feature space. Overall, strong DEGs constituted the primary discriminatory basis, while the inclusion of model-prioritized features contributed incremental improvements in multiclass tissue classification.

### 3.5 SHAP-based identification of tissue-specific predictive genes

We conducted SHAP (SHapley Additive exPlanations) analysis to interpret the decision-making process of the final RF-OvR model and to quantify the contribution of individual genes to tissue classification. To enhance biological interpretability, genes with unknown or uncharacterized biological functions (novel genes) were excluded from downstream analyses. Specifically, for genes that overlapped between tissues, we ensured unique tissue specificity by assigning each gene only to the single tissue where its mean absolute SHAP value (∣SHAP∣) rank was the highest.

**FIGURE 5:**
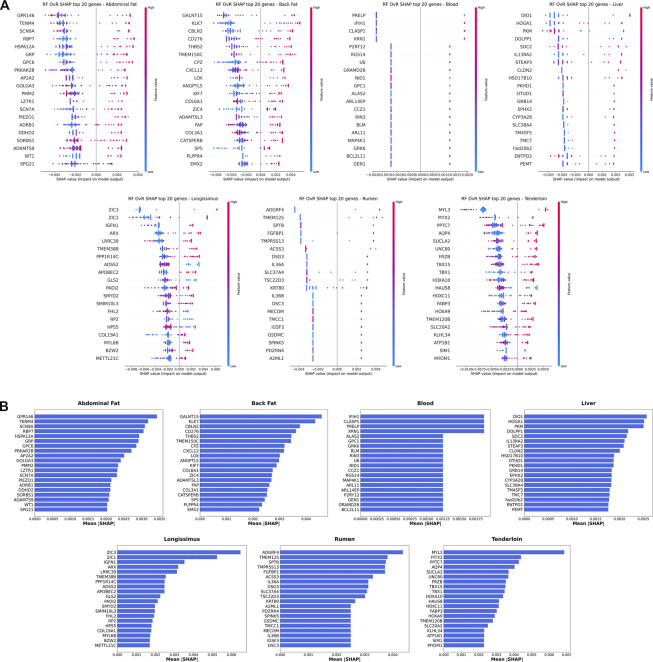
SHAP-based feature distribution and importance for top 20 predictive genes. **(A)** Beeswarm plots showing the distribution of SHAP values for the top 20 genes in each tissue-specific binary classifier. Each dot represents an individual sample, with color indicating gene expression level (red: high, blue: low). Positive SHAP values on the x-axis indicate an increased probability of classification into the corresponding target tissue. **(B)** Bar plots showing the mean absolute SHAP values of the same gene sets, ranked according to their contribution to model predictions.

This prioritization strategy allowed us to identify the top 20 genes for each tissue, resulting in a total of 140 tissue-specific predictive features. Figure 5 shows how SHAP values are distributed and how much each gene contributes to prediction. In the beeswarm plot (Figure 5A), genes with higher expression levels (shown in red) showed positive SHAP values across many tissue types. This suggests that high gene expression strongly supports correct tissue classification. In addition, the bar plots in Figure 5B display the mean absolute SHAP values. These results indicate that the selected genes explain the majority of the model’s decision-making process in each tissue classifier.

SHAP-based ranking analysis identified transcriptomic features contributed to tissue-specific classification in the Hanwoo training dataset. The agreement between SHAP-derived feature importance and tissue-specific expression patterns indicates stable predictive performance of the RF-OvR model.

### 3.6 Comparative analysis of SHAP-selected genes with differential expression genes

A strong agreement was observed between SHAP-based gene ranking and conventional DEG analysis (Table 5). Complete overlap between the two approaches was detected in Liver and Rumen, where all 20 SHAP-selected genes were also identified as DEGs. Abdominal Fat and Longissimus shared 19 overlapping genes, while Back Fat showed 18 overlapping genes. Blood and Tenderloin exhibited 16 overlapping genes each.

**TABLE 5:** Overlap between SHAP-prioritized top 20 genes and differentially expressed genes identified by One-vs-Rest analysis.

Tissue	Number of the overlapped genes	Overlapped genes		Non-overlapped genes
		Upregulated genes	Downregulated genes	
Abdominal fat	19	*GPR146, TENM4, SCN9A, RBP7, HSPA12A, GRP, GPC6, PRKAR2B, AP2A2, PMM2, LZTR1, SCN7A, PIEZO1, ADRB1, DDHD2, SORBS1, ADAMTS9, WT1, SPG21*	*-*	*GOLGA3*
Back fat	18	*GALNT15, KLK7, CBLN2, CD276, THBS2, TMEM150C, CPZ, CXCL12, LOX, ANGPTL5, KIF7, COL6A1, ADAMTSL3, FAP, COL3A1, CATSPERB, PLPPR4, EMX2*	*-*	*ZIC4, SP5*
Blood	16	*IFIH1, ALAS2, GRK6, BLM, RIN3, U6, RGS14, MAP4K1, ARL11, P2RY12, GEN1, GRAMD2B, BCL2L11*	*PRELP, GPC1, NID1*	*CLASP1, XRN1, CCZ1, ARL14EP*
Liver	20	*DIO1, HOGA1, DOLPP1, SDC2, IL13RA2, STEAP3, CLDN2, HSD17B10, PKHD1, GRB14, EPHX2, CYP3A28, SLC38A4, TM4SF5, TMC7, hsd20b2, PEMT*	*PKM, OTUD1, ENTPD2*	*-*
Longissimus	19	*ZIC3, ZIC1, IGFN1, ARX, LRRC39, TMEM38B, PPP1R14C, APOBEC2, PADI2, SMYD2, SMIM10L3, MYL6B, BZW2, METTL21C*	*ADSS2, GLS2, FHL2, RP2, HPS5*	*COL19A1*
Rumen	20	*ADGRF4, TMEM125, TMPRSS13, FGFBP1, IL36A, DSG3, KRT80, A2ML1, PDZRN4, SPINK5, GSDMC, MECOM, IL36B, IGSF3, DSC3*	*SPTB, ACSS3, SLC37A4, TSC22D3, TMCC1*	*-*
Tenderloin	16	*MYL3, PPTC7, SUCLA2, UNC80, FRZB, TBX15, HOXA10, FABP3, HOXA9, TMEM120B, SLC20A2, KLHL34, ATP1B1, SIM1, MYOM1*	*HAUS8*	*PITX2, AQP4, TBX1, HOXC11*

Differential expression analysis was conducted on the Hanwoo training dataset using a One-vs-Rest (OvR) comparison strategy. The results were used to assess the biological relevance of the top 20 SHAP-ranked genes for each tissue. DEGs were defined using a false discovery rate FDR-adjusted significance threshold (adjusted p-value <0.05) and an expression magnitude cutoff (|log2FC| > 1). In the One-vs-Rest (OvR) comparison, positive log2FC values indicate upregulation in the target tissue, whereas negative log2FC values indicate downregulation relative to all other tissues.

A substantial overlap was observed between the top 20 genes prioritized by SHAP and those identified by DEG analysis, demonstrating consistency between model-based feature selection and conventional statistical results. In contrast, a subset of SHAP-prioritized genes did not satisfy the DEG significance threshold. Despite this, these genes maintained relatively high SHAP importance values, indicating their involvement in RF-OvR–based tissue classification.

### 3.7 Characterization of tissue-level expression structure of SHAP-Prioritized genes

**FIGURE 6:**
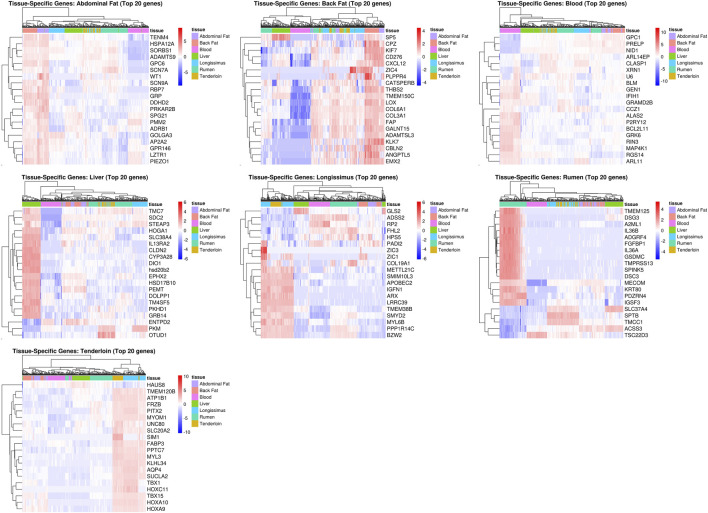
Integrated heatmap visualization of Tissue-Specific Expression Patterns of the Top 20 SHAP-Prioritized Genes. Heatmaps show the expression levels of the top 20 genes prioritized by SHAP for each tissue class (abdominal fat, back fat, blood, liver, longissimus, rumen, and tenderloin). Rows represent genes and columns represent samples. Gene expression values were scaled by z-score normalization. Hierarchical clustering was applied to both genes and samples based on Euclidean distance and complete linkage.

Genes prioritized by SHAP exhibited distinct tissue-dependent expression patterns across tissues, reflecting tissue-specific transcriptional characteristics (Figure 6). Hierarchical clustering analysis further showed that samples were predominantly grouped according to tissue type, with each tissue group forming a characteristic expression pattern. In contrast, partial overlap of expression patterns was observed for some genes among biologically related tissue groups, such as adipose and muscle tissues.

### 3.8 SHAP-based feature interaction analysis for tissue classification

**FIGURE 7:**
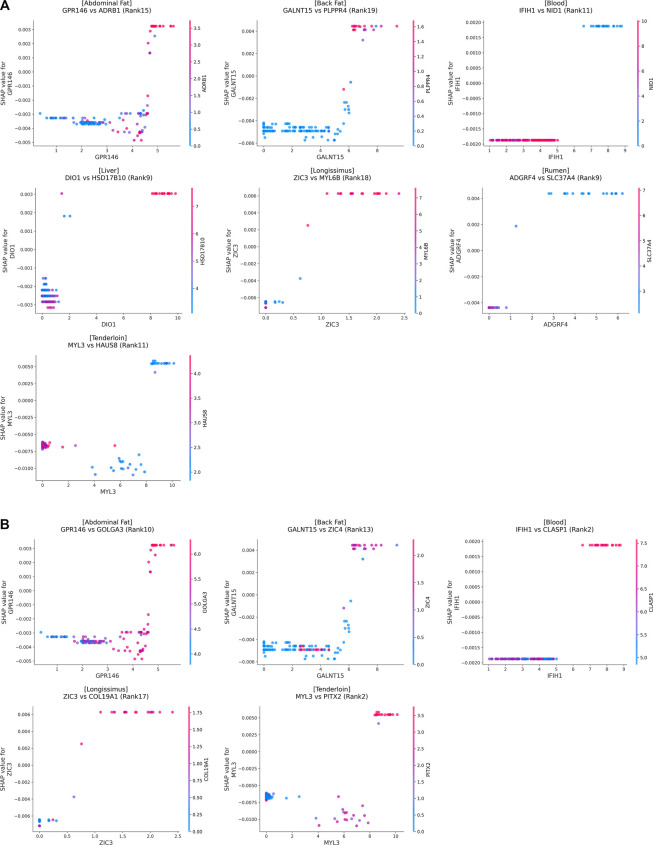
Representative SHAP dependence plots of SHAP-prioritized gene pairs across tissues. **(A)** SHAP dependence plots of the top-ranked (Rank 1) predictive gene for each tissue-specific RF-OvR classifier. The x-axis represents gene expression levels transformed as log2 (count+1), and the y-axis represents SHAP values indicating the contribution of the gene to tissue classification. Each point corresponds to an individual sample, and the color scale indicates the expression level of an auxiliary gene that satisfied the DEG criterion among the top 20 SHAP-ranked genes. **(B)** SHAP dependence plots of non-DEG genes that did not satisfy the differential expression criterion but showed high importance in SHAP analysis. Although these genes were not statistically significant in conventional differential expression analysis, they exhibited relatively high contributions in the model-based tissue classification process.

To further examine how SHAP-prioritized genes contribute to tissue classification, SHAP dependence plots were generated for representative gene pairs among the top 20 features for each tissue (Figure 7). Across tissues, the top-ranked gene exhibited expression-dependent variations in SHAP value distributions, indicating that its contribution to tissue classification differed across expression ranges rather than following a simple linear relationship. In addition, the SHAP contribution of the primary predictive gene varied according to the expression levels of auxiliary genes (Figure 7A), suggesting that the its classification relevance is influenced by the broader expression context of other features rather than by its expression level alone.

Several non-DEG genes that did not meet conventional differential expression criteria still exhibited measurable, tissue-specific SHAP contributions in dependence plots (Figure 7B). Although these genes were not statistically significant in univariate differential expression analysis, they contributed to the RF-OvR–based tissue classification process through their combinational expression patterns with other genes.

Together, these results demonstrate that both DEG and non-DEG genes prioritized by SHAP contribute in a complementary manner to the model-based tissue classification framework by capturing distinct aspects of the transcriptional decision structure.

## 4 Discussion

### 4.1 Machine learning model performance and classification strategy analysis

The RF-based OvR framework produced high classification accuracy across seven Hanwoo tissue types and maintained stable classification performance across seven Hanwoo tissue types, with a mean accuracy of 0.907 ± 0.004 and a mean AUC of 0.963 ± 0.004 under repeated balanced validation (Table 3). Consistent performance was further observed during independent external RNA-seq validation, indicating robust model behavior under varying sample compositions.

The OvR classification strategy has the advantage of defining tissue-specific decision boundaries by training independent binary classifiers for each tissue class. According to (Rifkin and Klautau, 2004), when combined with well-regularized binary classifiers, the One-vs-Rest (OvR) strategy can maintain stable learning behavior in high-dimensional settings where the number of features greatly exceeds the number of samples, while achieving classification performance comparable to more complex multiclass formulations. Consistent with these reports, the OvR-based RF model in this study outperformed multiclass RF and XGBoost models by 11%–18% in mean accuracy.

This property has been further supported in high-dimensional genomic and transcriptomic applications, where OvR-based regularized classifiers have been successfully applied to multiclass gene expression–based classification and feature selection tasks, including cancer subtype prediction and biomarker identification (Krishnapuram et al., 2005; Anand and Suganthan, 2009; Liu et al., 2012). Similar performance stability has also been reported in large-scale microarray and RNA-seq studies employing OvR-based learning strategies in high-dimensional gene expression settings (Statnikov et al., 2005; Way and Greene, 2018; Withnell et al., 2021). These studies support the suitability of OvR-based classifiers for reliably learning class-specific discriminative signals in high-dimensional biological data.

The proposed framework offers several advantages for transcriptomic data analysis. The model enables tissue-specific transcriptomic profiling while reducing the number of genes required for classification. Second, the use of SHAP-based interpretation enables identification of biologically meaningful candidate genes and improves model interpretability. Third, the framework is applicable to high-dimensional RNA-seq datasets and can be extended to other livestock species. These features make the proposed approach useful for tissue classification, biomarker discovery, gene prioritization, and functional interpretation in livestock transcriptomic studies.

To address class imbalance in the external validation dataset, repeated balanced subsampling was applied across tissue groups during model evaluation. This strategy reduced class-dependent prediction bias and improved the robustness of performance estimation, as evidenced by the consistently low standard deviation observed for the RF–OvR model across repeated balanced validations (±0.004, Table 3). These results indicate that the RF–OvR framework provides a stable and effective classification strategy for high-dimensional RNA-seq–based tissue discrimination.

### 4.2 Role of batch effect correction for data integration

**FIGURE 8:**
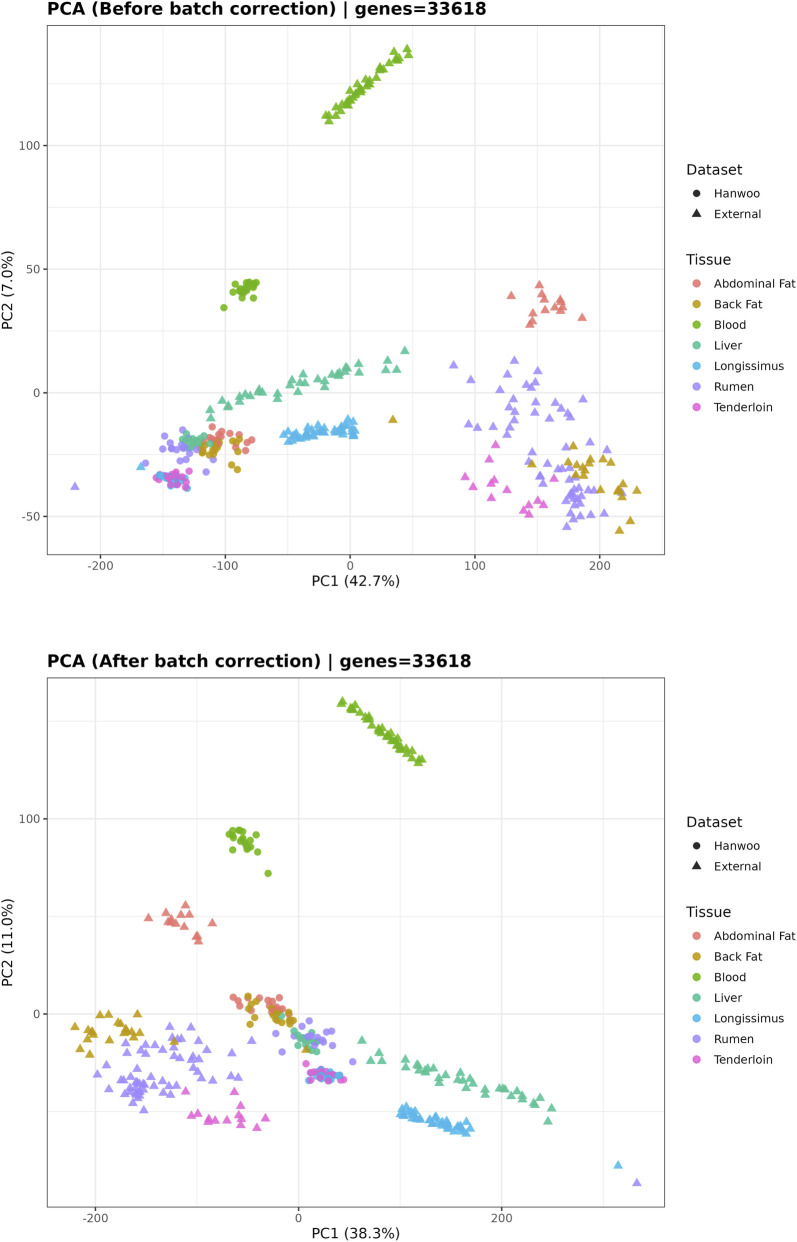
PCA visualization of batch effects before and after reference-batch ComBat correction. This figure presents principal component analysis (PCA) plots before and after batch effect correction. PCA was performed using 33,618 genes shared across datasets after excluding genes with zero expression or zero variance in the training set.

RNA-seq datasets generated under heterogeneous experimental and sequencing conditions frequently exhibit systematic technical variation that alters expression distribution patterns. Such technical artifacts can obscure biologically meaningful transcriptomic signals and negatively influence downstream classification performance. Consistent with this, principal component analysis (PCA) performed prior to batch correction revealed clear separation of samples according to dataset of origin rather than tissue type (Figure 8), indicating the presence of substantial batch effects between the training and external validation cohorts.

To mitigate this issue, we applied a reference-batch ComBat strategy using the training dataset as the reference batch. After batch correction, PCA showed that before batch correction, samples were primarily separated by dataset origin rather than tissue type, reflecting substantial batch effects between the training and external datasets. After applying reference-batch ComBat with the training dataset as the reference, dataset-driven variation in the principal components was markedly reduced. Although samples from the two datasets did not fully overlap, tissue-specific clustering became more evident, indicating that batch correction reduced technical variation while preserving biologically relevant transcriptional patterns.

Reference-based batch correction reduced distributional discrepancies while maintaining training data integrity, consistent with previous findings (Zhang, 2024).

As a result, the RF-OvR model maintained stable classification performance during external validation, supporting the role of batch effect correction in enabling robust cross-study generalization. This suggests that appropriate batch correction is a critical component for extending transcriptome-based tissue classification models to independent and heterogeneous RNA-seq datasets.

### 4.3 Complementary contribution of DEG and SHAP-Derived features

The comparative evaluation of feature selection strategies provided insight into the relative contributions of univariate statistical signals and multivariate model-derived signals to tissue classification. The strong performance of the Model 1 (DEG-only) indicates that major transcriptional differences between biologically distinct tissues are largely captured by highly differentially expressed genes. In particular, tissues such as Blood, Liver, and Rumen showed consistent separation, suggesting that large-scale expression shifts serve as dominant discriminative signals at the tissue level. The relatively stronger classification performance observed for Blood, Liver, and Rumen likely reflects the presence of more distinct tissue-specific gene expression profiles and physiological functions within the gene-level expression space used in the present study.

However, confusion matrix analysis revealed systematic limitations in distinguishing closely related tissues. Misclassification between Abdominal Fat and Back Fat, as well as between Longissimus and Tenderloin, persisted across models. These patterns indicate that DEG-based filtering alone does not sufficiently resolve fine-scale transcriptional variation within closely related tissue categories. Although transcriptional differences between Longissimus and Tenderloin are detectable, they appear to be concentrated within specific functional pathways rather than broadly distributed across the transcriptome (Yu et al., 2019). In these contexts, tissue divergence may be driven more by coordinated pathway activity and gene interaction structures than by large mean expression differences detectable through univariate DEG analysis (Zhao et al., 2025). Therefore, DEG-based filtering may not fully resolve subtle intra-muscle heterogeneity when underlying variation is network- or state-dependent rather than fold-change driven.

The lower performance of the Model 2 (SHAP-only and non-DEG) further highlights the importance of strong differential expression signals. Although SHAP-prioritized features contributed to the original RF–OvR decision process, they were not sufficient as an independent feature set. This suggests that multivariate interaction signals identified by SHAP operate in conjunction with strong differential expression signals, refining classification boundaries rather than replacing the primary discriminatory structure.

The integration of DEG and SHAP-derived features yielded the highest overall classification performance. The Model 3 (Union) improved separation between adipose tissues and reduced cross-classification involving Blood, indicating that statistically significant genes and model-derived features capture complementary aspects of transcriptional variation. This integrative approach increased classification stability without substantially expanding feature dimensionality, combining the strengths of both univariate and multivariate selection strategies.

Model 4, which used randomly selected genes with the same feature size, showed substantially lower and unstable classification performance compared with the other feature selection strategies. This result indicates that tissue classification performance does not arise from arbitrary gene subsets but depends on biologically informative gene selection. The poor performance of the random feature set supports the interpretation that both DEG-based signals and SHAP-prioritized genes capture meaningful transcriptomic structure relevant to tissue identity. Thus, Model 4 serves as a baseline control demonstrating that the observed predictive performance is not driven by feature number alone but by biologically relevant gene selection.

Relatively compact gene subsets were sufficient to achieve stable predictive performance. The DEG-only model maintained consistent accuracy with 133 genes, indicating that tissue-level transcriptional identity is concentrated within a limited set of informative features. This observation aligns with previous studies showing that a small number of highly informative genes can retain much of the classification structure in transcriptomic data, as demonstrated by ActiveSVM-based minimal gene set discovery (Chen et al., 2022).

Overall, strong differential expression signals provide the primary discriminatory structure for tissue classification, while SHAP-derived features contribute additional refinement, particularly in partially overlapping expression contexts. The strong performance of the DEG-only model suggests that tissue-level transcriptional identity in relatively homogeneous livestock datasets may be concentrated within a limited set of highly informative genes. For example, transcriptome analysis of multiple tissues from Nellore bulls representing extreme residual feed intake (RFI) groups identified a limited DEG signature sufficient to distinguish feed efficiency groups, suggesting that performance-related transcriptional differences were concentrated within a small set of informative genes (Zhang et al., 2019).

In contrast, in datasets with greater biological complexity and a large number of additive variables, multivariate or multimodal integration approaches may provide additional advantages. For example, the Multimodal CustOmics framework demonstrated that integrating complementary molecular features can improve classification performance by capturing nonlinear interactions across multiple data layers (Benkirane et al., 2025). Therefore, DEG-driven signatures may effectively capture dominant tissue-level transcriptional identity, whereas SHAP-based and multimodal approaches provide additional discriminatory power when transcriptomic differences are subtle or distributed across coordinated gene interactions.

However, the continued difficulty in separating closely related muscle tissues reflects intrinsic biological similarity that may not be fully resolved using bulk transcriptomic profiles. Accordingly, while tissue identity provided the major discriminatory structure at the overall dataset level, Longissimus and Tenderloin remained less distinctly separable than tissues such as Blood, Liver, and Rumen within the present framework. This limitation may also partly reflect the use of gene-level abundance rather than transcript-level abundance in the present study, which may have reduced sensitivity to isoform-level differences between closely related muscle tissues. Incorporating additional molecular layers or higher-resolution data may improve discrimination among fine-scale tissue subtypes in future analyses. Furthermore, it should be emphasized that the primary focus of this framework is to establish a robust strategy for tissue identity classification and to evaluate model interpretability rather than to identify direct markers of economic traits. Given the increasing application of machine learning approaches in transcriptomic research, another objective of the present study was to provide a useful framework for integrating machine learning–based feature prioritization with biological interpretation in Hanwoo cattle. In this context, the identification of tissue-specific markers was intended to define transcriptomic features that most strongly contribute to tissue discrimination within a machine learning framework and to explore how these genes may reflect the biological characteristics and functional properties of each tissue in Hanwoo cattle. Therefore, the prioritized genes should be interpreted primarily as tissue-discriminative transcriptomic signatures in the present dataset, rather than as direct indicators of economic traits or as absolute context-independent markers unaffected by physiological or developmental variation. Consequently, the prioritization of genes reflecting core physiological functions—such as immune signaling in blood or epithelial integrity in the rumen—demonstrates that the model successfully captured the fundamental biological identity of each tissue, thereby supporting the biological interpretability of the proposed classification approach.

### 4.4 Model interpretation and biological validity through SHAP analysis

In this study, SHAP analysis was performed to interpret the predictive mechanism of the RF-OvR model and to assess the biological validity of the tissue classification results. The SHAP-based feature importance analysis showed that the SHAP-prioritized genes identified for each tissue were in high consistency with tissue-specific biological functions reported in previous studies. This suggests that the model performs predictions based on biological transcriptomic patterns, not only statistical patterns.

To further support biological interpretation, we expanded the functional annotation of the SHAP-prioritized genes across tissues. Genes identified in adipose tissues were mainly associated with lipid metabolism and adipocyte differentiation, whereas blood-specific genes were related to immune and hematopoietic functions. Liver-associated genes were primarily involved in metabolic regulation, rumen genes reflected epithelial structure and barrier function, and muscle tissue genes were linked to myogenic regulation, mitochondrial activity, and muscle fiber characteristics. These findings indicate that the identified genes are consistent with known tissue-specific biological roles.

#### 4.4.1 Abdominal fat

In abdominal fat, genes directly associated with lipid metabolism and energy homeostasis were identified as key SHAP predictive features. *PRKAR2B*, which encodes the RIIβ regulatory subunit of protein kinase A, plays a critical role in adipose tissue metabolism and energy homeostasis (Cummings et al., 1996). Mouse knockout studies have demonstrated that disruption of *PRKAR2B* results in a lean phenotype and increased energy expenditure, indicating a protective role against diet-induced obesity (Cummings et al., 1996; Czyzyk et al., 2008). In human adipose tissue, reduced *PRKAR2B* expression has been associated with obesity-related metabolic dysfunction, including decreased PKA activity and impaired lipolytic responses in both subcutaneous and visceral fat depots (Mantovani et al., 2009). Notably, although *PRKAR2B* shows clear differential expression across tissues, its high SHAP importance suggests that its predictive contribution is further shaped by expression-dependent and context-specific patterns rather than mean expression differences alone. In addition, *RBP7* is an adipose-enriched gene that shows elevated expression in adipose tissues and has been reported to be associated with adipocyte differentiation and retinoid-related lipid metabolic regulation pathways (Ahn et al., 2018). These results demonstrate that the RF-OvR model effectively learned molecular signals consistent with the metabolic characteristics of abdominal fat, reinforcing both the interpretability and biological validity of the tissue classification framework.

#### 4.4.2 Back Fat

In back fat, genes associated with adipocyte differentiation, tissue microenvironment regulation, and extracellular matrix (ECM) remodeling were identified as key SHAP predictive features. *GALNT15* has been reported as a gene induced during adipogenesis in preadipocyte models (Takahashi et al., 2024), and structural variants near *GALNT15* are known to be associated with fat deposition traits in bovine populations (Upadhyay et al., 2021). Furthermore, *CXCL12*, which encodes the chemokine CXCL12, has been reported as a chemokine secreted by mature adipocytes that induces macrophage influx and inflammatory responses in adipose tissue (Li et al., 2023). This suggests the model utilizes transcriptomic patterns reflecting the structural and immunological characteristics of subcutaneous adipose tissue.

#### 4.4.3 Blood

In blood, genes directly associated with immune responses and hematopoietic function were identified as key SHAP-predictive features. *IFIH1*, which encodes a key factor mediator of innate immune signaling that recognizes viral RNA (Najm et al., 2024), while *ALAS2*, which encodes the rate-limiting enzyme in the erythrocyte-specific heme biosynthesis pathway, plays an essential role in hematopoiesis (Chiabrando et al., 2014). The high SHAP contribution of these genes demonstrates that the model performed classification based on immune and hematopoietic transcriptomic patterns consistent with the functional characteristics of blood tissue.

While SHAP-prioritized genes for blood were predominantly associated with immune and hematopoietic functions, the model also captured systemic metabolic signals. Features such as *CHCHD10*, *PAFAH2*, and *PHIP*, which are involved in energy metabolism and insulin signaling, were identified in the extended ranking. This suggests that immune and hematopoietic transcriptional programs contributed more directly to blood tissue classification in the present dataset, whereas the blood transcriptome also retained signals related to broader physiological and energy-associated processes. This pattern may reflect the fact that immune-related features provided more immediate discriminatory information within the current classification framework.

#### 4.4.4 Liver

In liver, genes directly associated with liver-specific metabolic functions were identified as key SHAP features. *DIO1* is highly expressed in the liver and its transcription is regulated by hepatic transcription factors including FOXA1, FOXA2, and LXRα, which directly bind to the hDIO1 promoter and modulate its activity (Sakane et al., 2017). *HOGA1*, which encodes an enzyme expressed in the liver and kidneys that participates in regulating oxaloacetate metabolism in the gluconeogenesis pathway (Huang et al., 2019). *HSD17B10*, which encodes a mitochondrial-localized enzyme involved in steroid and lipid-related metabolic processes. It has been reported to be highly expressed in metabolically active tissues such as the liver and to participate in mitochondrial phase I–related metabolic pathways and redox-associated reactions (Sahadevan et al., 2015; He et al., 2025), while *GRB14*, which encodes a highly expressed insulin signaling inhibitor in the liver with reported functions in regulating hepatic lipid metabolism and hepatocyte proliferation (Popineau et al., 2016; Morzyglod et al., 2017). Furthermore, *EPHX2* is known to be highly expressed in the liver and is an enzyme involved in regulating lipid metabolism and stress responses (Qin et al., 2019; Mello et al., 2021), while *CYP3A28* and *SLC38A4* are reported as liver-specific genes closely associated with xenobiotic metabolism and amino acid transport functions in the bovine liver (Giantin et al., 2019; Liu et al., 2025). These results demonstrate that the model effectively utilizes transcriptome signals consistent with the central metabolic functions of liver tissue.

#### 4.4.5 Rumen

In rumen, genes associated with epithelial structure formation, barrier function, and immune regulation were identified as key SHAP features. *ADGRF4* was reported as a hub gene in rumen-related co-expression modules in yak rumen transcriptome analysis (Liu et al., 2023), suggesting a potential central role in rumen epithelial function regulatory networks. *TMPRSS13*, which encodes a membrane-associated serine protease whose expression increases in the adult rumen, has been reported to be associated with epithelial barrier function (Pan et al., 2020; Pokhrel and Jiang, 2024). *IL36A* and *IL36B*, which encode cytokines involved in regulating immune and inflammatory responses in rumen epithelium, known to play crucial roles in ruminal acidosis and rumen development (Clark et al., 2017; Gholizade et al., 2020). Epithelial structure-related genes *DSG3*, *DSC3*, and *KRT80* have also been reported as key markers involved in maintaining the mechanical stability and barrier function of ruminal epithelium (Clark et al., 2017; Li et al., 2019; Ji et al., 2021), and *A2ML1* has been utilized as an RNA-seq validation marker as a representative transcript stably expressed in rumen tissue (Wang et al., 2021; Liu et al., 2023). These results demonstrate that the model effectively learned the molecular patterns reflecting the epithelial-specific structure and immune environment of rumen tissue.

#### 4.4.6 Longissimus

In Longissimus, genes associated with myogenic regulation, myofiber structural organization, and muscle growth were identified as key SHAP features. In particular, myogenic regulatory factors *ZIC1* and *ZIC3*, which encode transcription factors regulating early myogenic differentiation processes through Myf5 activation. Comparative transcriptome studies in cattle also indicate their relatively high expression levels in Longissimus tissue (Yu et al., 2019; Sheet et al., 2024). In addition, *ARX*, encoding a homeobox transcription factor, has been reported as a regulatory factor promoting embryonic myogenesis by cooperating with the MyoD-MEF2C-Myogenin pathway, suggesting its association with muscle lineage-specific differentiation regulation (Sheet et al., 2024).

Genes associated with sarcomeric organization were also identified as key SHAP features, including *LRRC39*, encodes a muscle-specific M-band protein involved in sarcomere stability and mechanosensing (Will et al., 2010; Ehrlich et al., 2020). This demonstrates that the model utilizes molecular signals reflecting the structural characteristics of Longissimus tissue.

Genes associated with muscle growth and post-translational regulation also ranked as top SHAP features. *METTL21C*, which encodes a lysine methyltransferase regulating myoblast proliferation through methylation of the IGF2BP1 protein (Wang et al., 2023), while *PPP1R14C* was proposed as a candidate gene for muscle development regulation in an integrated analysis of Longissimus muscle transcriptomes and chromatin accessibility (Miao et al., 2021). Furthermore, *PADI2*, which encodes an enzyme mediating protein citrullination, has been reported as a Longissimus-enriched gene in both cattle and pig transcriptome studies, with associations also suggested to muscle remodeling and meat quality traits (Guo et al., 2019; Yu et al., 2019; Sheet et al., 2024). These results suggest that the RF-OvR model utilizes transcriptional signals directly associated with muscle development, structural maintenance, and growth regulation during the classification of Longissimus tissue.

#### 4.4.7 Tenderloin

In tenderloin, genes associated with muscle fiber type specification, oxidative metabolic activity, and mitochondrial function were identified as key SHAP features. The transcription factor encoded by *TBX15* has been reported to regulate skeletal muscle fiber type identity and to control oxidative metabolic programs (Lee et al., 2015). A study analyzing Hanwoo muscle fiber composition reported that tenderloin tissue has a higher proportion of oxidative muscle fibers compared to other regions (Cho et al., 2024). In addition, *TBX1*, which encodes a transcription regulator associated with myogenic progenitor differentiation and energy metabolism, has been reported to be associated with increased expression in tenderloin and intramuscular fat deposition traits (Motohashi et al., 2019; Sheet et al., 2024).

Genes related to mitochondrial function and energy metabolism were also derived as Tenderloin-specific SHAP features. *PPTC7*, which encodes a mitochondrial protein phosphatase essential for mitochondrial protein import, metabolic regulation, and organelle biogenesis, has been reported to contribute to the maintenance of mitochondrial function and energy metabolism (Niemi et al., 2019). Furthermore, *SUCLA2*, which encodes an enzyme in the TCA cycle, was reported to show higher protein expression levels in Tenderloin compared to Longissimus, suggesting an association with differences in mitochondrial activity between muscle sites (Ramanathan et al., 2021).

Genes related to muscle contraction and cellular homeostasis were also identified as key features. *MYL3* encodes the myosin light chain protein, which is directly involved in regulating muscle contraction, and has been reported as a molecular marker associated with meat quality and tissue texture in various livestock species (Xie et al., 2022; Sheet et al., 2024). Furthermore, *AQP4*, encoding a skeletal muscle-enriched water channel, has been reported to contribute to intracellular water transport, cell volume homeostasis, and fatigue resistance in muscle fibers (Aslesh et al., 2023). *MYOM1* encodes the myomesin-1 protein, reported to play a key role in thick filament alignment and force transmission within myofibers (Ropka-Molik et al., 2021). These results suggest that the model distinguishes Tenderloin muscle by using transcriptomic signals that reflect metabolic specialization, fiber-type composition, and mitochondrial energy metabolism.

SHAP-based feature attribution analysis indicated that genes with high contribution scores were aligned with biologically meaningful transcriptional patterns reported in previous transcriptomic studies. Similar consistency between model-derived feature importance and biologically interpretable structures has been reported in tree-based explanation frameworks and interpretable transcriptome modeling approaches (Way and Greene, 2018; Lundberg et al., 2020; Sidak et al., 2022). Importantly, SHAP dependence plot analysis demonstrated that the contributions of key genes were not proportional to their expression levels but instead followed non-linear, threshold-like patterns shaped by multigene expression contexts. These findings indicate that prediction outputs were driven by biologically relevant expression signals rather than dataset-specific statistical artifacts.

### 4.5 SHAP-based visualization of tissue-specific transcriptomic patterns

The heatmap analysis based on SHAP-prioritized top 20 genes for each tissue revealed that the selected gene sets in each tissue tended to form distinct expression patterns centered on the corresponding tissue samples (Figure 6). This visualization provides a structural validation of the SHAP-based feature prioritization, demonstrating that the selected genes are not only important for classification but also exhibit coherent tissue-specific expression profiles at the transcriptome level. In particular, major tissues such as fat (Abdominal Fat, Back Fat), liver, muscle (Longissimus, Tenderloin), and rumen formed distinct sample clusters in the hierarchical clustering results. These clustering patterns indicate that the tissue-specific transcriptomic signals learned by the RF-OvR model are structurally reflected in gene expression levels, rather than arising from isolated gene-level effects.

For abdominal and back fat tissues, each SHAP-prioritized top 20 genes displayed distinct expression distributions despite belonging to the same adipose tissue category. The observed separation reflects depot-specific metabolic and regulatory differences at the transcriptome level. Molecular-level variation encoded in gene expression profiles enabled the RF-OvR classifier to discriminate adipose subtypes beyond simple tissue category boundaries.

In liver samples, metabolism related genes consistently exhibited high expression levels and formed a liver tissue-specific cluster. In skeletal muscle tissues (Longissimus and Tenderloin), genes associated with muscular structure and function showed expression patterns clearly separated from those of non-muscle tissues. Genes associated with epithelial structure formation and barrier regulation showed high SHAP contribution values in rumen samples, generating expression profiles characteristic of digestive tissue physiology.

In contrast to differential expression analysis, machine learning models often identify gene sets that differ from those selected by explainability methods, while achieving comparable classification performance using coordinated contributions from numerous lower-ranked features, suggesting that individually non-significant genes can still contribute to discrimination in a multivariate context (Bontonou et al., 2025). This distinction highlights a fundamental limitation of single gene centered statistical approaches, as machine learning–based models infer tissue-specific classification boundaries by modeling high-dimensional and multivariate expression relationships rather than relying on isolated gene level signals (Alharbi and Vakanski, 2023).

### 4.6 Applicability and industrial implications of the study

The present findings indicate that transcriptome-based machine learning classification frameworks can be feasibly extended to both livestock research applications and industrial production environments. Previous studies have reported that integrating transcriptomic prediction models with explainable artificial intelligence enables not only accurate tissue classification but also biologically interpretable gene-level insights (Zhao et al., 2022). By combining the RF-OvR classification model with SHAP-based interpretation, we achieved robust tissue discrimination performance while providing interpretability at the gene level. This combination addresses a key limitation of conventional black-box models and supports the practical deployment of transcriptomic classifiers in real-world biological and production environments, where model transparency and reliability are essential.

From an applied standpoint, the tissue-specific gene signatures identified in this study provide a basis for developing molecular markers that support meat quality assessment and carcass trait evaluation. Integrated transcriptomic and metabolomic studies have reported that important meat quality traits, such as intramuscular fat content, tenderness, and water-holding capacity, are closely associated with specific gene expression patterns and metabolic pathways (Zhang et al., 2025). Consistent with these findings, the transcriptomic differences observed between abdominal and back fat, as well as muscle-specific expression patterns, suggest molecular characteristics that may be associated with economically important production traits.

Furthermore, the SHAP-prioritized genes provide candidate molecular markers that may support downstream applications in breeding and livestock improvement pipelines. Omics-based information has been reported to be utilized as supplementary data to enhance the prediction accuracy of complex traits such as productivity, fat deposition, and feed efficiency in livestock improvement programs (Chakraborty et al., 2022). Compared with univariate DEG-based filtering strategies, machine learning–based methods capture multivariate expression relationships and gene interaction patterns, allowing a more realistic representation of the genetic complexity underlying polygenic traits. This strategy enables the proposed framework to supplement conventional single marker selection strategies.

SHAP dependence plot analysis indicates that the RF-OvR model captures tissue classification through coordinated multi-gene expression patterns, consistent with the ability of tree-based ensemble models to learn complex feature interactions (Breiman, 2001; Statnikov et al., 2008). In contrast to conventional DEG analysis, which focuses on univariate mean expression differences, SHAP-based feature attribution reflects gene contributions to model decision boundaries, indicating that these approaches provide complementary perspectives for interpreting transcriptomic data (Guyon and Elisseeff, 2003).

While our interpretation primarily focused on tissue-specific functions such as epithelial barrier integrity in the rumen, it is plausible that the multivariate signals captured by the RF-OvR model also reflect inter-tissue metabolic interactions. Because the present framework was constructed as a One-vs-Rest tissue classification model, our interpretation was centered on the most direct tissue-discriminative functions represented by the prioritized genes in each classifier, rather than on cross-tissue regulatory relationships. Accordingly, the current analysis was not designed to directly infer metabolic coupling between tissues, although the possibility of such biological relationships cannot be excluded. For instance, in ruminants, the metabolic flux of volatile fatty acids (VFAs) from the rumen to the systemic circulation plays a pivotal role in regulating lipid synthesis and overall meat production performance. Future integrative modeling incorporating multi-tissue or multi-omics data may help further elucidate these coordinated transcriptional signatures.

Across most tissues, Rank 1 genes exhibited threshold-like behavior in the SHAP dependence plots, where SHAP values changed abruptly within specific expression ranges. This pattern indicates that tissue-specific transcriptomic signals influence classification decisions in a nonlinear manner rather than through simple linear increases in expression, consistent with the ability of tree-based models to approximate nonlinear decision boundaries in high-dimensional settings (Statnikov et al., 2008). Furthermore, the vertical dispersion of SHAP values within similar expression intervals may reflect feature interaction signals, in line with the SHAP interaction framework (Lundberg and Lee, 2017a; Lundberg et al., 2018).

Several genes with limited univariate differential expression signals nonetheless contributed substantially to classification performance through coordinated multigene expression patterns. In this context, the results of this study suggest that univariate DEG-based filtering alone struggles to fully capture classification-related signals inherent in multivariate expression structures, supporting the necessity of machine learning-based multivariate analysis strategies in transcriptomic data interpretation.

Collectively, SHAP-based interpretability approaches mitigate the black-box nature of conventional machine learning models by quantitatively decomposing and visualizing tissue-specific signals learned within the model at the sample level. Recent studies have demonstrated that SHAP-based visualization frameworks improve the transparency and reliability of model interpretation by providing consistent local and global feature attribution across supervised learning models (Ponce-Bobadilla et al., 2024).

### 4.7 Limitations and future research directions

The machine learning-based transcriptomic classification and interpretation framework proposed in this study effectively captured tissue-specific molecular signals by learning complex multivariate interaction structures among genes. Nevertheless, several limitations should be considered. Although the training dataset was balanced across tissues and suitable for initial model construction, the limited sample size in some tissue groups during external validation may constrain the full assessment of model generalization performance. Previous studies have also reported that the composition and sample distribution of independent validation datasets are important factors in evaluating the generalization performance of RNA-seq-based machine learning models. (Chen et al., 2021).

Another point that should be considered is the heterogeneous paternal background of the animals included in the training dataset. Because sire background can influence offspring transcriptomic patterns, residual genetic heterogeneity cannot be completely excluded in the present multi-sire dataset. In addition, transcriptomic profiles may reflect not only tissue identity itself but also the biological and physiological conditions under which each tissue is represented. However, our additional confounding-factor assessment showed that sire background did not produce clear clustering patterns in tissue-wise PCA and was not significant in any tissue in tissue-wise PERMANOVA, suggesting that paternal heterogeneity did not act as a major global confounding factor in the current dataset. Nevertheless, compared with a single-lineage design, the use of animals derived from multiple sires may still introduce subtle background variation. Therefore, the tissue-specific transcriptomic classifiers identified in this study should be interpreted as tissue-discriminative patterns observed within the biological context represented in the present dataset, rather than as entirely context-independent markers. Future studies using larger lineage-balanced or single-lineage animal groups, together with more tightly controlled physiological conditions, would be valuable for further validating the robustness of these tissue-specific transcriptomic classifiers.

Another methodological consideration is that the present study was conducted using gene-level expression quantified by featureCounts rather than transcript-level or isoform-level abundance. We chose this strategy to maintain a stable and comparable feature space across the Hanwoo training dataset and heterogeneous public RNA-seq datasets used for external validation, and to enable direct comparison between DEG-based statistical\ signals and SHAP-based model-derived feature prioritization within the same analytical framework. However, transcript-level quantification may capture tissue-specific isoform usage with higher resolution, and the use of gene-level aggregated counts may therefore have limited sensitivity for distinguishing closely related tissues, particularly the two muscle tissues. In addition, the log_2_ (count +1) transformation applied to gene-level abundance does not preserve isoform-specific expression patterns, and transcript-level abundance measures such as TPM may be more suitable for resolving transcript isoform variation in tissue-specific analyses. Future studies incorporating transcript-level quantification approaches, including alignment-free tools such as Kallisto or Salmon, may provide additional biological resolution and improve tissue discrimination, while also helping to clarify whether isoform-level variation contributes to the relatively stronger separation observed for tissues such as Blood, Liver, and Rumen.

Feature attribution derived from machine learning models reflects decision-boundary formation mechanisms that differ conceptually from conventional DEG-based statistical testing. While DEG analysis focuses on evaluating the expression changes of individual genes through univariate statistical tests based on average expression differences, the machine learning approach learns classification boundaries by utilizing the combinatorial expression patterns and interaction structures of multiple genes. Indeed, low concordance between machine learning–based importance rankings and DEG-based gene rankings has been reported, suggesting these approaches may reflect distinct biological signals (Liu et al., 2022). These methodological differences indicate that integrated analytical strategies combining statistical and model-based interpretation approaches are required for comprehensive transcriptomic analysis.

Several strategies may help address the current limitations of this study. First, future work using larger and more diverse independent datasets would improve the robustness and generalizability of the proposed framework. Second, inclusion of additional tissues and broader biological variation may allow a more comprehensive evaluation of tissue-specific transcriptomic patterns, and comparative assessment of gradient boosting and deep learning-based frameworks may further improve the capacity to capture additional nonlinear patterns in high-dimensional biological datasets (Sartori et al., 2025). Third, a systematic comparison with advanced architectures, such as Graph Neural Networks (GNNs) that capture gene-regulatory networks or Transformer-based models for high-dimensional embedding, may further refine feature selection and predictive accuracy beyond tree-based ensembles. In addition, integration of transcriptomic data with other omics layers, such as proteomic functional translation or metabolomics for phenotypic validation, may enhance biological interpretation of tissue-associated gene sets. Finally, conducting *in vitro* functional assays such as CRISPR-based gene silencing or over-expression in bovine cell lines will be crucial to empirically validate the biological relevance of the SHAP-prioritized candidate markers identified in this study.

## 5 Conclusion

Hanwoo cattle transcriptome data were used to construct a tissue classification model, and this study examined the biological relevance of the prediction results. The Random Forest One-vs-Rest (OvR) classifier evaluated independent RNA-seq datasets generated under different experimental conditions and resulted similar classification performance across data sources.

SHAP-based interpretation revealed that tissue classification was driven by biologically meaningful gene expression patterns, including both tissue-specific differentially expressed genes and additional genes contributing through coordinated multigene expression structures. Accordingly, SHAP-based prioritization in this study should be interpreted primarily as identifying genes that contribute most strongly to tissue discrimination within the model, rather than as a direct ranking of biological importance. Biologically relevant but lower-ranked genes may still emerge through complementary analyses such as DEG-based selection, pathway interpretation, and literature-supported functional annotation. Feature-set comparison further demonstrated that strong differentially expressed genes constituted the primary discriminatory basis for tissue classification, while integration with SHAP-derived features improved predictive performance, indicating that univariate statistical signals and multivariate model-derived signals provide complementary information in transcriptome-based classification. Importantly, stable performance was achieved using relatively compact gene subsets, suggesting that tissue-level transcriptional identity is concentrated within a limited number of informative features rather than being driven by feature quantity alone. The structured comparison between univariate DEG filtering and model-derived SHAP prioritization further demonstrates that transcriptomic classification is supported by a hierarchical signal structure. Strong differential expression defines the primary tissue identity, whereas coordinated multigene interaction patterns refine class boundaries. This integrative framework provides a reproducible strategy for distinguishing statistical significance from predictive relevance in high-dimensional transcriptomic data.

These results suggest that integrating multigene-based machine learning analysis with conventional univariate statistical analysis can provide a more effective and biologically grounded strategy for transcriptome data interpretation. The tissue-specific genes identified in this study can be used in various applications, including beef quality evaluation, marker-assisted selection, and livestock genomic research. Moreover, this analytical approach can be applied to transcriptome data from other livestock species and extended by integrating additional omics datasets. Furthermore, functional experiments and additional validation will help refine the biological interpretation and practical applicability of these findings.

## Data Availability

The original contributions presented in the study are publicly available. The raw RNA-seq datasets generated for this study have been deposited to the NCBI Sequence Read Archive (SRA) under BioProject accession number PRJNA1426915.
